# Effects of inbreeding and other systematic effects on fertility of Black Forest Draught horses in Germany

**DOI:** 10.1186/s13028-017-0338-4

**Published:** 2017-10-18

**Authors:** Maarit Müller-Unterberg, Sandra Wallmann, Ottmar Distl

**Affiliations:** Institute for Animal Breeding and Genetics, University of Veterinary Medicine Hannover (Foundation), Bünteweg 17p, 30559 Hannover, Germany

**Keywords:** Black Forest Draught horse, Fertility, Foaling rate, Horse, Inbreeding

## Abstract

**Background:**

The Black Forest Draught horse (BFDH) is an endangered German coldblood breed with its origin in the area of the Black Forest in South Germany. In this retrospective study, the influence of the inbreeding coefficient on foaling rates was investigated using records from ten breeding seasons. Due to the small population size of BFDH, the level of inbreeding is increasing and may have an effect on foaling rates.The data of the present study included all coverings reported for 1024 BFDH mares in the years 2001–2009. These mares were covered by 32 BFDH stallions from the State Stud Marbach. Data from 4534 estrus cycles was used to calculate per cycle foaling rate (CFR). Pedigree data contained all studbook data up to the foundation of the breed as early as 1836. The level of inbreeding of the mare, stallion and expected foal along with other systematic effects on CFR were analysed using a generalized linear mixed model approach. Stallion was employed as a random effect. Systematic fixed effects were month of mating, mating type, age of the mare and stallion, reproductive status of the mare and stallion line of the mare. Inbreeding coefficients of the stallion, mare and expected foal were modelled as linear covariates.

**Results:**

The average CFR was 40.9%. The mean inbreeding coefficients of the mares, stallions and expected foals were 7.46, 7.70 and 9.66%. Mating type, age of the mare, reproductive status of the mare and stallion line of the mare had a significant effect.

**Conclusions:**

The results showed that the mating type, stallion line of the mare, sire, age and reproductive status of the mare exerted the largest influences on CFR in BFDH. Inbreeding coefficients of the stallion, mare and expected foal were not significantly related with CFR.

**Electronic supplementary material:**

The online version of this article (doi:10.1186/s13028-017-0338-4) contains supplementary material, which is available to authorized users.

## Background

The Black Forest Draught horse (BFDH) is considered as an endangered horse breed mainly bred in the area of the Black Forest in Baden-Wuerttemberg region, located in southern Germany [1]. The predominant coat colour of the BFDH is dark chestnut with a light mane. Anecdotically, these horses are believed to be descendants of the “Wälderpferd”, a local horse breed kept by farmers in the Black Forest. Several other horse breeds influenced the BFDH in the last centuries. A studbook was established as early as 1896. Genealogically the Black Forest horse belongs to the Noric Horse group [2].

Due to the increasing motorization of agriculture in the 1950s, the BFDH population size decreased to 159 mares and only two stallion-lines [1]. These two stallion lines were founded by the Belgian coldblood stallion Deutschritter (D-line) and the Noric stallion Milan (M-line). From the 1970s, the Freiberg stallion Dayan (F-line), the Schleswig coldblood stallion Varus (V-line) and the Noric stallions Reith-Nero (R-line), Wirts-Diamant (W-line) and Riff-Vulkan (K-line) were introduced to establish new five stallion lines and to broaden the genetic diversity [1, 3]. These measures have prevented a further erosion of the BFDH population. In the year 2013, 979 mares and 79 stallions were registered. Nevertheless, BFDH is still considered as an endangered breed [3].

The genetic bottleneck in the 1950s led to a decrease of the effective number of founders. For the birth years between 2000 and 2010, 29 founders were identified for BFDH [4]. A small effective number of founders with an unbalanced contribution of ancestors can reduce genetic variation and promote inbreeding. An unbalanced distribution of the genetic contributions of ancestors could be observed in the BFDH population between 2000 and 2010 resulting in average inbreeding coefficients per year at 7.8–9.8% [4].

Previous reports indicate that fertility of horses might be influenced by inbreeding [5–10] besides many other factors [11–28]. The objectives of the present study were to determine coefficients of inbreeding for mares, stallions and expected foals and to quantify the effect of inbreeding coefficients on foaling rates in BFDH. In order to account for environmental and stallion-associated effects, month of mating, mating type, age of mare and stallion, reproductive status of the mare, stallion line of the mare and stallion were regarded in the analysis.

## Methods

The data included all reported matings of BFDH mares from the years 2001 to 2009 in Baden-Wuerttemberg. All BFDH stallions were kept at the State Stud Marbach. Reproductive records for each mare included the date of birth, each covering date, the type of mating and the identity and date of birth of the respective stallion. An oestrus cycle was defined through the intervals between two consecutive matings in a breeding season. Matings within intervals of less than 6 days were considered as one cycle. The outcome of an oestrus cycle was counted as successful if the birth of a foal was registered and the respective oestrus cycle was the last one registered within the respective breeding season. An oestrus cycle was considered as unsuccessful either when a further mating after the respective oestrus cycle was reported or when no foaling after the last reported oestrus cycle within the respective breeding season was registered. Based on this definition, per cycle foaling rate (CFR) was calculated for each oestrus cycle. All coverings were within the breeding season from February to August.

The data contained 32 BFDH stallions and 1024 mares. Number of stallions and mares per year along with number of cycles and foalings are given in Table 1.

**Table 1:** Number of stallions, mares, cycles and foals and foaling rates per season observed for the years 2001–2009

Year	Stallions (n)	Mares (n)	Cycles (n)	Foals (n)	Per cycle foaling rate (%)	Foaling rate per season (%)
2001	17	294	342	138	40.35	46.94
2002	18	337	419	178	42.48	52.82
2003	19	355	462	188	40.69	52.96
2004	19	326	457	186	40.70	57.06
2005	22	361	528	222	42.05	61.50
2006	25	356	543	211	38.86	59.27
2007	24	348	569	251	44.11	72.13
2008	26	343	621	255	41.06	74.34
2009	24	293	593	223	37.61	76.11
Total	32	1024	4534	1852	40.85	61.46

A multifactorial analysis of variance was performed to test the influence of inbreeding coefficients and further systematic effects on CFR in BFDH. Analysis was performed using a generalized linear mixed model (GLIMMIX procedure) of SAS, version 9.4, with a binomial distribution and a logit link function.

η_ijklmnopqrs_ = μ + MONTH_i_ + TYPE_j_ + STATUS_k_ + M_AGE_l_ + S_AGE_m_ + b_1_ × M_INBR_n_ + b_2_× S_INBR_o_ + b_3_ × O_INBR_p_ + LINE_q_ + stallion_r_ + e_ijklmnopqrs_ with η = dependent variable, logit for the per cycle foaling rate (CFR); μ = model dependent constant; MONTH_i_ = fixed effect of the month of first mating (i = 1,…,5; 1 = February–March, 2 = April, 3 = May, 4 = June, 5 = July–August); TYPE_j_ = type of breeding (j = 1,…,4; 1 = natural mating, 2 = artificial insemination with fresh semen at the stallion’s home station (on-site AI), 3 = artificial insemination with fresh semen transported to another station, 4 = artificial insemination with frozen semen); STATUS_k_ = fixed effect of the reproductive status of the mare (k = 1,…,3; 1 = mare with a foal from a mating in the preceding season, 2 = mated in the preceding year but no foaling was reported, 3 = not mated in the preceding year); M_AGE_l_ = fixed effect of age class of the mare at mating (l = 1,…,4; 1 = 2–5 years, 2 = 6–8 years, 3 = 9–12 years, 4 = > 12 years); S_AGE_m_ = fixed effect of age class of the stallion at mating (m = 1,…,4; 1 = 2–5 years, 2 = 6–8 years, 3 = 9–12 years, 4 = > 12 years); b_1_, b_2_ and b_3_ = linear regression coefficients for inbreeding coefficients of the mare, stallion and expected foal on CFR; M_INBR_n_ = inbreeding coefficient of the mare; S_INBR_o_ = inbreeding coefficient of the stallion; O_INBR_p_ = inbreeding coefficient of the expected foal; LINE_q_ = fixed effect of the stallion line of the mare (q = 1,…,7; 1 = stallion line D, 2 = stallion line F, 3 = stallion line K, 4 = stallion line M, 5 = stallion line R, 6 = stallion line V, 7 = stallion line W); stallion_r_ = random effect of the permanent environment of the stallion (r = 1–32) and e_ijklmnopqrs_ = residual error.

A parameterization of inbreeding coefficients in classes was done to account for non-linear relationships with CFR with M_INBR_n_ = fixed effect of the inbreeding class of the mare (n = 1,…,4; 1 = 0–6%, 2 = 7–8%, 3 = 9–10%, 4 = > 10%); S_INBR_o_ = fixed effect of the inbreeding class of the stallion (o = 1,…,4; 1 = 0–6%, 2 = 7–8%, 3 = 9–10%, 4 = > 10%); O_INBR_p_ = fixed effect of the inbreeding class r of the expected foal (p = 1,…,4; 1 = 0–7%, 2 = 8–9%, 3 = 10–12%, 4 = > 12%);

The median age of mares at covering was 9.0 years. The distribution of the age classes for mares remained stable across the ten breeding seasons. Age of stallions ranged from 3 to 25 years with a median age of 9.3 years. Stallions with an age of 2 to 5 years were employed in 38.8% of all coverings and the age group with an age > 12 years in 18.4% of the coverings. Stallion lines were defined according to their founder sire [2, 4]. In this data set, seven different stallion lines with three to eight sires for each line were distinguished. The pedigree used for calculating the inbreeding coefficients included data of 2900 stallions and 4158 mares which constituted all registered horses born between 1836 and 2010. Inbreeding coefficients were calculated for each mare, stallion and expected foal using ENDOG software [29]. The average generation equivalent of the BFDH population between 2000 and 2010 was 7.2. Grouping of inbreeding classes of expected foals was adapted to a more uniform distribution of number per class resulting in a slight difference in the threshold between classes. Inbreeding classes among mares, stallions and expected progeny were independently distributed allowing a simultaneous parameterization in a model. Estimated effects did not change when only inbreeding class of mare or stallion or expected foal was regarded. A random mare effect could not be regarded in the model due to missing convergence of the equations.

## Results

A total of 1852 foals were born alive between 2001 and 2009, resulting in an overall foaling rate per season of 61.46% and a CFR of 40.85%. The mean inbreeding coefficient was 7.46, 7.70 and 9.66% in mares, stallions and expected foals. The average inbreeding coefficient per year steadily increased from 2001 to 2008 with maximum values at 7.93, 8.13 and 10.32% in mares, stallions and expected foals. In 2009, a slight decrease of inbreeding coefficients was seen in all three groups (Additional file 1).

The mean CFR across all breeding seasons was 40.9%. A time trend of CFR with year was not obvious. The average of CFR for individual stallions ranged from 29.7 to 56.3%, which seemed indicative for large differences among stallion fertility.

Analysis of variance showed significant effects for the age of the mare at covering, status of the mare, mating type and stallion line of the mare (Table 2). Age and status of the mare had the largest influence on CFR among the effects considered. In mares and expected foals, a slight non-significant tendency for declining CFR with increasing inbreeding coefficients was found (Table 3). A similar tendency showed the analysis of classes of inbreeding coefficients for mares (Additional file 2) and expected foals (Additional file 3). CFR were highest in expected foals with inbreeding coefficients at 0 to 7% (Additional file 3).

**Table 2:** Results of the analysis of variance for the fixed effects using a generalized linear model with the stallion as random effect

Effect	DF	F value	P value
Inbreeding mare	1	0.21	0.6435
Inbreeding stallion	1	0.02	0.8934
Inbreeding expected foal	1	0.80	0.3705
Month of covering	4	2.43	0.0453
Age of stallion	3	0.94	0.4204
Age of mare	3	18.15	< 0.0001
Status of mare	2	29.19	< 0.0001
Mating type	3	8.83	< 0.0001
Stallion line of mare	6	3.39	0.0189

**Table 3:** Estimated linear regression coefficients with their standard errors (SE) of inbreeding coefficients in percent of the expected foal, stallion and the mare on per cycle foaling rate as 0/1-trait

Linear regression coefficient for	Regression coefficient ± SE
Expected foal	− 0.01139 ± 0.01272
Stallion	0.002201 ± 0.01643
Mare	− 0.00499 ± 0.01079

With increasing age of the mares the CFR were continuously decreasing (Table 4). Young mares showed the highest CFR. Mares with a foal at foot had the highest CFR (Table 4). Differences among the different mating types were larger in comparison to any other systematic fixed effect. Due to the low number of matings other than natural services the 95% confidence intervals of the odds ratios were large and thus, F-values smaller. CFR were highest for natural service matings, followed by transported semen and on-site insemination (Table 4). Stallion lines significantly differed among each other, with the largest differences among stallion lines R and K.

**Table 4:** Estimated odds ratios with their 95% confidence limits of the per cycle foaling rate by classes for the fixed effects of age of the mare at covering, status of the mare before covering, mating type and month of covering

Levels of the fixed effects	Number of observations	Odds ratio	95%-CI
Age of mare (years)			
2–5	1179	1.98	1.65–2.39
6–8	1136	1.85	1.54–2.22
9–12	1079	1.50	1.25–1.80
> 12	1054	1.00	
Status of mare			
Foaled	1119	1.76	1.50–2.06
Barren	1616	1.02	0.88–1.18
Not mated	1799	1.00	
Mating type			
Natural mating	3887	4.01	1.73–9.29
On-site AI	425	2.05	0.88–4.78
Transported semen	169	3.24	1.34–7.82
Frozen semen	45	1.00	
Month of covering			
February–March	354	0.83	0.62–1.13
April	1026	1.21	0.96–1.52
May	1508	1.14	0.92–1.42
June	1150	1.15	0.91–1.44
July–August	496	1.00	
Stallion line of mare			
D	1023	0.88	0.71–1.10
F	186	1.16	0.82–1.64
K	43	2.79	1.40–5.56
M	1373	1.03	0.83–1.26
R	1212	0.79	0.63–0.97
V	89	1.15	0.72–1.83
W	608	1.00	

The permanent environmental variance of the stallion comprised 1.89 ± 1.62% (P = 0.12) of the total variance. Due to the large standard error, the random effect of the stallion was not significant.

In addition, we tested an extended model with year of covering and book size per stallion. Year of covering and book size did only show very small effects on CFR in BFDH. Odds ratios among years were at 0.96–1.27 and non-significant. Book sizes per stallion and year ranged from 1 to 75 mares with an average of 18.2 mares/year. Only three stallions had a book size with more than 50 mares per season. Odds ratios were at 0.90–1.00 and far from significance. Due to the very small effects on CFR and P values > 0.8, we did not follow up this extended model.

Threshold models including an animal effect with a relationship matrix did not converge and thus, it was not possible to employ a statistical model accounting for a more complex data structure.

## Discussion

The seasonal foaling rate of BFDH in 2001 to 2009 is comparable with that of the coldblooded breeds in Finland [9, 21, 22] and France [7] with 52 to 66.5% foaling rates per season. The coldblooded horses Breton, Comtois, Ardenne, Percheron and Boulonnais in France had lower seasonal foaling rates than French warmblood or Thoroughbred mares [7], and the coldblooded Finnhorses achieved lower seasonal foaling rates than the Finnish Standardbred [9]. Studies of Thoroughbreds indicated foaling rates of 43–57% per cycle [12, 13] and 52–78% per season [11–13, 16, 18, 19, 24, 26–28]. In Standardbreds, foaling rates were 29–58% per cycle [5] and 50–76% per season [5, 9, 22].

In agreement with the present results, in Thoroughbreds no significant effects of inbreeding coefficients of the mares on fertility were found [8]. However, the level of inbreeding in this latter report was below 1%. In French horses, an increase of the inbreeding coefficients of the mares by 1% led to a decline in mares’ reproductive productivity by 0.5–1% [7]. For the inbreeding coefficients of the sires, opposite results were obtained. The authors suggest that this could be due to better care concerning the reproduction of inbred stallions.

In Standardbred Pacers with an average degree of inbreeding of 7.4% a negative effect of inbreeding of potential offspring on foaling rates was reported [5], but contrary results were found in Finnish Trotters with an average degree of inbreeding of 10.3%. In Finnish Standardbreds foaling rates markedly decreased when inbreeding coefficients of the expected foals were higher than 15%, whereas in Finnhorses foaling rates were lowest at inbreeding coefficients of the expected foals at 4.0–9.99% [9].

In Norwegian Trotters a significant effect of inbreeding of potential offspring on early pregnancy loss rate had been reported. However, the influence of inbreeding on foaling rate was not significant [6].

Inbreeding depression of fertility may be expected in horses [6, 11]. A non-significant result may be due to the recording of fertility as an all-or-none trait and an unsatisfactory registration practice of horse breeders. Consequences could be slightly overestimated foaling rates and thus underestimated effects of inbreeding coefficients on fertility traits. In general, differences in experimental design, animals examined and the method used to estimate the degree of inbreeding complicate a comparison between studies. For instance, the effect of inbreeding coefficients was often studied as linear regression and the fertility was defined as seasonal foaling rate in most cases. In this study, inbreeding was regarded as a linear regression. An additional analysis using classes for inbreeding coefficients gave similar results. In addition, CFR use the information from all cycles with coverings and should reflect the fertility status more accurate than seasonal rates. Summarizing the results for the effect of inbreeding on reproductive performance in BFDH, there is no evidence for significant negative effects. However, the limited size of the examined population and low numbers of matings between highly inbred individuals should be taken into consideration.

Nevertheless, there may be other reasons for differing foaling rates. For the Finnhorse population and the French breeds a traditional management and a lack in foal registrations are documented [7, 22]. Studies of Thoroughbred mares [3–7, 9, 11, 20, 32] and the Hanoverian Warmblood [17, 25] demonstrate the important role of management factors, too. This is especially true for the time after the introduction of ultrasonography for pregnancy detection and the use of prostaglandin F2α and human chorionic gonadotropin [13, 28].

One of the examined environmental factors in this study is the mating type. Foaling rates after artificial insemination are significantly lower than with other mating types. Natural cover resulted in very high foaling rates in Thoroughbreds [13, 27]. Seminal plasma is supposed to have an immunosuppressive effect in the uterus of the mare and stimulates endogenous prostaglandin synthesis [30]. The subsequent inflammation of the myometrium leads to uterine contractions that help to remove inflammatory products before the embryo reaches the uterus lumen [31]. Furthermore, seminal plasma proteins are crucial for the fertilization process [32].

The mares showed a constant decline in CFR with higher ages which is not in agreement with previous reports. A significant decline of fertility measures became mostly evident in middle-aged mares [12, 13, 16, 18, 22, 27] from 9 to 14 years. The reason for declining reproductive productivity in mares aged > 9 to14 years might be progressive degenerative changes in the endometrium and increasing susceptibility to infection [33, 34]. In the present study, the youngest mares had highest CFR suggesting an early start of degenerative endometrial changes. The age of stallions did not influence CFR and an age-related decrease was not obvious. Similarly, no effect on foaling rates had been shown in Thoroughbreds and Icelandic horses [14, 24]. Ewert et al. [16] observed a decrease of pregnancy rates for matings with Thoroughbred stallions > 16 years of age. Langlois and Blouin [7] pointed out that stallions can only be used up to an age of 20 years if their yearly number of mated mares is limited progressively.

Mares with a foal in the preceding breeding season had higher CFR than barren or mares with no matings in agreement with previous reports [7, 9, 21].

Conception rates were highest in the months April, May and June. Similar results have been obtained for Thoroughbred mares in Sweden [18] and Germany [16]. Mares foaling later in the season have less usable cycles to get pregnant again [7]. In addition, the accumulation of subfertile mares that were covered repeatedly without success has a negative effect on fertility at the end of the breeding season as well [16, 17, 26].

Mares descending from sires of the M-line were superior in CFR compared with sires of the D-line and R-line. Sires of lines F, K and V were presented only by a small number of mares, but had highest CFR. As the R-line and M-line were founded by Noric stallions, a general breed effect through the stallion line seems very unlikely. The effect of the stallion line may indicate an influence of an additive maternal effect on CFR. Heritabilities for foaling rate per season were at 0.01 in Standardbred [9], 0.03 in Finnhorses [25] and 0.12 in Polish warmblood horses [35].

## Conclusions

Mare’s age, status of the mare, stallion line of the mare and mating type exerted the largest influences on CFR. Increasing inbreeding coefficients in mares and expected foals were related with a slight tendency to decreasing CFR. Control of inbreeding in BFDH is necessary to avoid negative effects on fertility in further generations.

## Additional files

**Additional file 1.** The figure shows the distribution of the inbreeding coefficient of the stallions, mares and expected foals throughout the years.

**Additional file 2.** Estimated odds ratios with their 95% confidence limits of the per cycle foaling rate by classes for the inbreeding coefficients of the stallion and the mare. P-values for inbreeding coefficients of the stallion and the mare were 0.6930 and 0.2264.

**Additional file 3.** Estimated odds ratios with their 95% confidence limits of the per cycle foaling rate by classes of the inbreeding coefficients of the expected foal. P value for the inbreeding coefficient of the expected foal was 0.3326.

## Abbreviations

BFDH: Black Forest Draught horse
CFR: per cycle foaling rate

## References

[1] Weber M (2001). Stand und Weiterentwicklung der Schwarzwälder Kaltblutzucht in Baden-Württemberg zur Jahrhundertwende 2000, 2001: entwicklung der Kaltblutzucht seit,1947bis heute. Festschrift zum.

[2] Aberle K, Wrede J, Distl O (2003). Analyse der Populationsstruktur des Schwarzwälder Kaltblutpferdes. Berl Münch Tierärztl Wochenschr.

[3] Domestic Animal Diversity Information System (DAD-IS, global system for domestic animal diversity), Food and Agriculture Organization of the United Nations (FAO), Rome. http://www.fao.org/dad-is. Accessed June 2017.

[4] Müller-Unterberg M, Wallmann S, Distl O (2013). Schätzung der genetischen Diversität der Schwarzwälder Kaltblutpopulation anhand von Pedigreedaten. Züchtungskunde.

[5] Cothran EG, MacCluer JW, Weitkamp LR, Pfennig DW, Boyce AJ (1984). Inbreeding and reproductive performance in Standardbred horses. J Hered.

[6] Klemetsdal G, Johnson M (1989). Effect of inbreeding on fertility in Norwegian trotter. Livest Prod Sci..

[7] Langlois B, Blouin C (2004). Statistical analysis of some factors affecting the number of horse births in France. Reprod Nutr Dev.

[8] Mahon GAT, Cunningham EP (1982). Inbreeding and the inheritance of fertility in the Thoroughbred mare. Livest Prod Sci.

[9] Sairanen J, Nivola K, Katila T, Virtala AM, Ojala M (2009). Effects of inbreeding and other genetic components on equine fertility. Animal.

[10] van Eldik P, van der Waaij EH, Ducro B, Kooper AW, Stout TAE, Colenbrander B (2006). Possible negative effects of inbreeding on semen quality in Shetland pony stallions. Theriogenology.

[11] Baker CB, Little TV, McDowell KJ (1993). The live foaling rate per cycle in mares. Equine Vet J.

[12] Bosh S, Powell D, Shelton B, Zent W (2009). Reproductive performance measures among Thoroughbred mares in central Kentucky, during the 2004 mating season. Equine Vet J.

[13] Brück I, Anderson GA, Hyland JH (1993). Reproductive performance of Thoroughbred mares on six commercial stud farms. Aust Vet J.

[14] Davies Morel MCG, Gunnarsson V (2000). A survey of the fertility of Icelandic stallions. Anim Reprod Sci..

[15] Dyrmundsson OR (1994). Reproduction of Icelandic horses with special reference to seasonal sexual activity. Icelandic Agric Sci.

[16] Ewert M, Böröcz J, Uphaus H, Oldenhof H, Distl H, Sieme H (2013). Einflussfaktoren auf die Trächtigkeitsrate in der deutschen Vollblutzucht. Tierärztliche Praxis Großtiere.

[17] Hamann H, Mertens U, Sieme H, Klug E, Distl O (2005). Einfluss des Besamungsmanagements auf Fruchtbarkeitsmerkmale in der Population des Hannoverschen Warmbluts. Züchtungskunde.

[18] Hemberg E, Lundeheim N, Einarsson S (2004). Reproductive performance of Thoroughbred mares in Sweden. Reprod Dom Anim.

[19] Hevia ML, Quiles AJ, Fuentes F, Gonzalo C (1994). Reproductive performance of Thoroughbred horses in Spain. J Equine Vet Sci.

[20] Hugason KM, Arnason Th, Jonmundsson JV (1985). A note on the fertility and some demographical parameters of Icelandic toelter horses. Livest Prod Sci.

[21] Katila T, Nivola K, Reilas T, Sairanen J, Peltonen T, Virtala A-M (2010). Factors affecting reproductive performance of horses. Pferdeheilkunde.

[22] Katila T, Reilas T, Nivola K, Peltonen T, Virtala A-M (2010). 15-year survey of reproductive efficiency of Standardbred and Finnhorse trotters in Finland—descriptive results. Acta Vet Scand.

[23] McDowell K (2002). Reproductive success in broodmares – Part 1. Equine Disease Quarterly..

[24] McDowell K, Powell DG, Baker CB (1992). Effect of booksize and age of mare and stallion on foaling rates in Thoroughbred horses. J Equine Vet Sci..

[25] Merkt H, Jacobs K-O, Klug E, Aukes E. An analysis of stallion fertility rates (foals born alive) from the breeding documents of the Landgestüt Celle over a 158-year period. J Reprod Fertil. 1979;Suppl. 27:73–77. 383990

[26] Merkt H, Klug E, Jochle W (2000). Reproduction management in the German Thoroughbred breeding industry. J. Equine Vet Sci.

[27] Morris LHA, Allen WR (2002). Reproductive efficiency of intensively managed Thoroughbred mares in Newmarket. Equine Vet J.

[28] Schulman ML, Marlow CHB, Nurton JP (2003). A survey of reproductive success in South African Thoroughbred horse breeding from 1975 to 1999. J S Afr Vet Ass..

[29] Gutierrez JP, Goyache F (2005). A note on ENDOG: a computer program for analysing pedigree information. J Anim Breed Genet.

[30] Katila T (2001). Sperm-uterine interactions: a review. Anim Reprod Sci.

[31] Troedsson MHT, Loset K, Alghamdi D, Dahms B, Crabo BG (2001). Interaction between equine semen and the endometrium: the inflammatory response to semen. Anim Reprod Sci.

[32] Töpfer-Petersen E, Waberski D, Hess O, Bellair S, Schamboy A, Ekhlasihundrieser M (1998). Bedeutung des Seminalplasmas für die Befruchtung - ein kurzer Überblick. Tierärtzl Umschau.

[33] Bracher V, Gerstenberg C, Allen WR (1997). Der Einfluss von Endometrose (degenerativer Endometriumserkrankungen) auf Fruchtbarkeit, Plazentation und fotale Entwicklung beim Pferd. Pferdeheilkunde.

[34] Carnevale EM, Ginther OJ (1992). Relationships of age to uterine function and reproductive efficiency in mares. Theriogenology.

[35] Wolc A, Torzynski G, Szwaczkowski T (2009). Genetic effects on reproductive traits in Warmblood horses. Can J Anim Sci.

