# Supplementary material for: Effects of inbreeding and other systematic effects on fertility of Black Forest Draught horses in Germany
Source: Acta Vet Scand. 2017 Oct 18;59:70. doi: 10.1186/s13028-017-0338-4 (PMC5648486; doi:10.1186/s13028-017-0338-4)

Additional file 1

The figure shows the distribution of the inbreeding coefficient of the stallions, mares and expected foals throughout the years

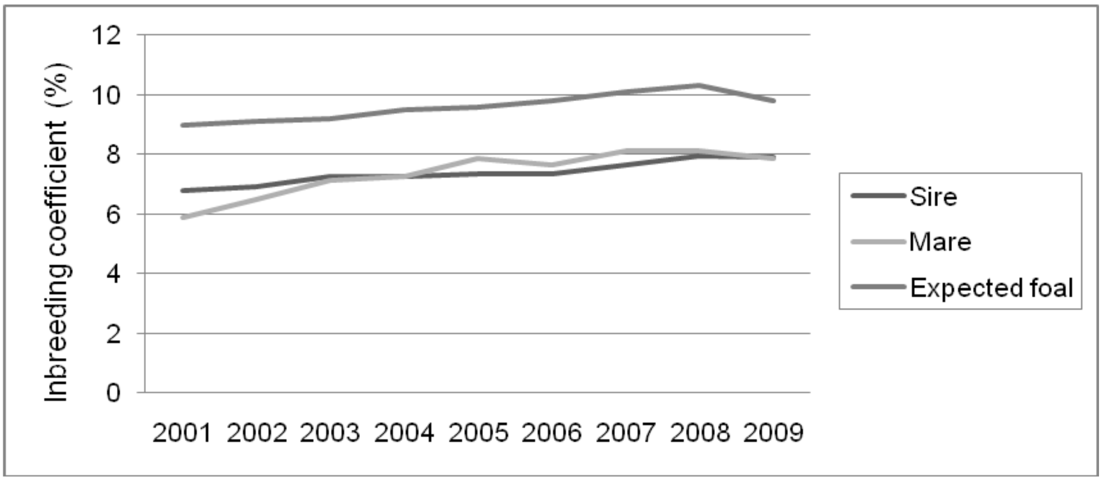

Supplement: Supplementary file 1 — Additional file 1. The figure shows the distribution of the inbreeding coefficient of the stallions, mares and expected foals throughout the years. [file 13028_2017_338_MOESM1_ESM.pdf]
