# Supplementary material for: Effects of inbreeding and other systematic effects on fertility of Black Forest Draught horses in Germany
Source: Acta Vet Scand. 2017 Oct 18;59:70. doi: 10.1186/s13028-017-0338-4 (PMC5648486; doi:10.1186/s13028-017-0338-4)
Supplement: Supplementary file 2 — Additional file 2. Estimated odds ratios with their 95% confidence limits of the per cycle foaling rate by classes for the inbreeding coefficients of the stallion and the mare. P-values for inbreeding coefficients of the stallion and the mare were 0.6930 and 0.2264. [file 13028_2017_338_MOESM2_ESM.pdf]

Additional file 2 Estimated odds ratios with their 95% confidence limits of the per cycle foaling rate by classes for the inbreeding coefficients of the stallion and the mare. P-values for inbreeding coefficients of the stallion and the mare were 0.6930 and 0.2264.

Inbreeding coefficient (%)	Stallion			Mare		
	Number of observations	Odds ratio	95%-CI	Number of observations	Odds ratio	95%-CI
0 - 6	1549	0.99	0.77 -1.27	1419	1.12	0.91 -1.37
6 – 8	722	1.10	0.84 - 1.43	1173	1.09	0.90 - 1.32
8 – 10	121	0.94	0.74 - 1.21	1119	0.94	0.78 - 1.14
>10	1042	1.00		823	1.00	
