# Supplementary material for: Effects of inbreeding and other systematic effects on fertility of Black Forest Draught horses in Germany
Source: Acta Vet Scand. 2017 Oct 18;59:70. doi: 10.1186/s13028-017-0338-4 (PMC5648486; doi:10.1186/s13028-017-0338-4)
Supplement: Supplementary file 3 — Additional file 3. Estimated odds ratios with their 95% confidence limits of the per cycle foaling rate by classes of the inbreeding coefficients of the expected foal. P value for the inbreeding coefficient of the expected foal was 0.3326. [file 13028_2017_338_MOESM3_ESM.pdf]

Additional file 3 Estimated odds ratios with their 95% confidence limits of the per cycle foaling rate by classes of the inbreeding coefficients of the expected foal. P-value for the inbreeding coefficient of the expected foal was 0.3326.

Inbreeding coefficient (%)	Number of observations	Odds ratio	95%-CI
0 – 7	935	1.15	0.92 - 1.43
7 – 9	1206	0.96	0.80 - 1.15
9 – 12	1248	1.02	0.86 - 1.21
>12	1145	1.00	
